# In Situ Formation of CoS_2_ Hollow Nanoboxes via Ion-Exchange for High-Performance Microwave Absorption

**DOI:** 10.3390/nano12162876

**Published:** 2022-08-21

**Authors:** Dongwei Xu, Huanhuan Guo, Feifan Zhang, Yanmei Wu, Xiaoqin Guo, Yumei Ren, Desheng Feng

**Affiliations:** School of Material Science and Engineering, Henan Key Laboratory of Aeronautical Materials and Application Technology, Zhengzhou University of Aeronautics, Zhengzhou 450046, China

**Keywords:** CoS_2_, hollow nanoboxes, ion-exchange, microwave absorption

## Abstract

Hollow nanoboxes structure have raised great attention as microwave absorption materials on account of their ultralow density and large specific area. By introducing an adjustable interior cavity structure, the dielectric loss and microwave absorption performance were affected by the tunable complex permittivity and impedance matching was improved. In our study, hollow CoS_2_ nanoboxes with designable interspaces were successfully fabricated based on the surfactant-assisted solution method and followed by an in situ ion-exchange process. The structure, elemental compositions and morphology of the products were characterized by XRD, XPS, EDX, SEM and TEM, respectively. In addition, microwave absorption performance and the intrinsic mechanism are investigated in-depth. The paraffin-based composites with 20 wt.% filling contents exhibited superior microwave absorption capacities in view of both maximum reflection loss value (*RLmax*, −54.48 dB) and effective absorption bandwidth (*EAB*, below −10 dB, 6.0 GHz), which can be ascribed to unique hollow structure and good impedance matching. With these considerations in mind, this study provides a reference for the construction of high-performance microwave absorbers with unique hollow structure.

## 1. Introduction

The electromagnetic radiation generated by the extensive use of new electronic devices such as household electrical appliances, wireless communication and advanced military radars has led to the deterioration of the electromagnetic environment, posing a serious threat to human health and safety, normal operation of equipment as well as national defense security, which has aroused worldwide concern [1,2,3,4,5]. Electromagnetic shielding and electromagnetic wave absorption have long been considered as two representative measures to mitigate or resist the adverse effects of redundant electromagnetic waves. The former realizes a shielding effect by strong reflection of incident electromagnetic wave, and the latter is based on the absorption and attenuation of the incident electromagnetic wave into heat energy or other forms to achieve the purpose of consuming electromagnetic energy. Due to its different mechanisms, electromagnetic absorption has gradually evolved into the main means of electromagnetic pollution prevention because of its ideal sustainability [3,6,7,8].

Recently, transition metal sulfides (TMSs), due to their excellent electric and magnetic properties, have been used in a wide range of applications, such as in lithium-ion batteries [9,10], supercapacitors [11,12] and microwave absorption [13,14,15]. Among them, cobalt disulphide (CoS_2_) nanoparticles, because of their high thermal stability and good electronic conductivity, show special advantages in microwave absorption fields [16]. Furthermore, many studies indicate that CoS_2_ has a half-metallic nature and unique magnetic properties, which could enhance the absorption performance. Recently, hollow or porous nanostructure materials have aroused widespread research interest in the microwave absorption field, due to their multi-reflection and scattering as well as the improved impedance matching. Therefore, hollow carbon spheres [17], hollow graphene microspheres [18], yolk-shell microspheres [19], tubular carbon nanofibers [20] and 3D graphene foams/aerogels [21,22] are being developed. Compared to reported research, transition metal sulfides (TMSs) with hollow or porous structure show lower pristine density and better corrosion resistance. Meanwhile, because of their unique structural characteristics and intrinsic performance, the special porous or hollow structure with tunable shell thickness and interior void endow materials with an interior cavity, low density and large surface area, which could overcome the above shortcomings. Until now, many hollow structures (spheres, boxes and nanotubes) have been successfully fabricated based on soft- or hard-template methods, such as Co_1−x_S hollow spheres [23], porous Co_9_S_8_ nanotubes [24], hierarchical hollow CuS@CoS_2_ nanoboxes [25] and paramagnetic CoS_2_@MoS_2_ core-shell composites [14]. However, complex synthesis processes and subsequent removal of templates have limited their applications.

In our work, we first adopt the surfactant-assisted solution method and in situ ion-exchange process in the presence of CS_2_ to successfully fabricate CoS_2_ hollow nanoboxes with tunable interiors and shell thickness. As absorbents, the paraffin-based composites with 20 wt.% filler loading displayed excellent microwave absorbing ability, which is considered by the modification of electronic and geometric effects. Moreover, the current studies provided extensive impetus for the rational design and controllable synthesis of well-defined hollow nanoboxes with excellent absorption performance.

## 2. Materials

All reagents were at analytic reagent grade and used as received without additional purification. Cobalt nitrate (Co(NO_3_)_2_·6H_2_O), 2-Methylimidazole and Hexadecyl trimethyl ammonium Bromide (CTAB) was purchased from Aladdin Corporation. Anhydrous ethanol was purchased from Sinopharm Chemical Reagent Co., Ltd. (Shanghai, China).

### 2.1. Synthesis of Solid ZIF-67 Precursor and CoS_2_ Hollow Nanoboxes

**Figure 1:**
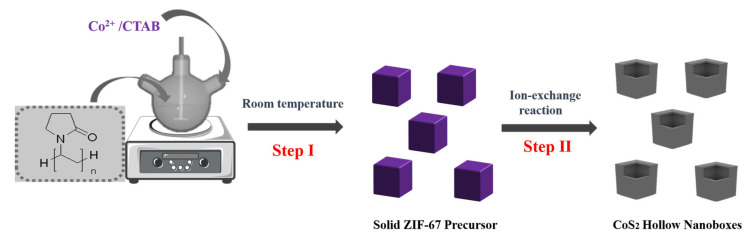
Schematic illustration for the preparation of CoS_2_ hollow nanoboxes.

In this typical synthesis process, Co(NO_3_)_2_·6H_2_O (0.292 g) and CTAB (0.01 g) were dissolved into 10 mL deionized water under ultrasonic stirring to form clear solution (defined as A solution). 2-methylimidazole (2-MIM, 4.54 g) were dissolved into 70 mL deionized water under stirring to form solution B. After that, solution A was injected into solution B rapidly and stirred continuously for 2 h. After the reaction, the purple products were centrifuged, washed three times with water and redispersed in 60 mL anhydrous ethanol containing 0.3 mL CS_2_. Finally, the mixture was sealed into a 100 mL stainless steel autoclave and kept at 140 °C for 8 h. The autoclave was cooled down to room temperature naturally and the black precipitate was washed with ethanol several times. The final products were collected by centrifugation and dried in a hot oven at 70 °C for 12 h. The synthetic procedure is depicted in Figure 1, where the highly uniform solid ZIF-67 precursor was obtained, using CS_2_ as the sulfur source. The solid ZIF-67 precursor was successfully transformed into CoS_2_ nanoboxes. Due to the different diffusion rate of inward diffusion of vulcanizing agent and outward diffusion of metal ions, the vulcanizing agent reacted with metal ions on the surface of the solid ZIF-67 precursor and began to produce a core-shell structure. With the extension of vulcanization time, the ion exchange reaction was completed and the hollow CoS_2_ nanoboxes with unique composition and structure were obtained.

### 2.2. Characterization

The crystal structures of all samples were recorded by X-ray powder diffraction (XRD) using Cu Kα radiation (λ = 0.15418 nm). The morphology and inner structure information were investigated by field-emission scanning electron microscope (FESEM/EDS, XFlash 5030, Bruker, Germany), transmission electron microscope (TEM, Tecnai F30, FEI Inc., Hillsborough, CA, USA), high resolution TEM as well as scanning TEM (STEM) images and elemental mapping analyses. The chemical states and elemental composition of the samples were determined through X-ray photoelectron spectroscopy (XPS). The electromagnetic parameters of the products were measured by a network analyzer (Agilent 8720 ET, Santa Clara County, CA, USA) in the frequency range of 1–18 GHz via a coaxial ring method.

## 3. Results and Discussion

**Figure 2:**
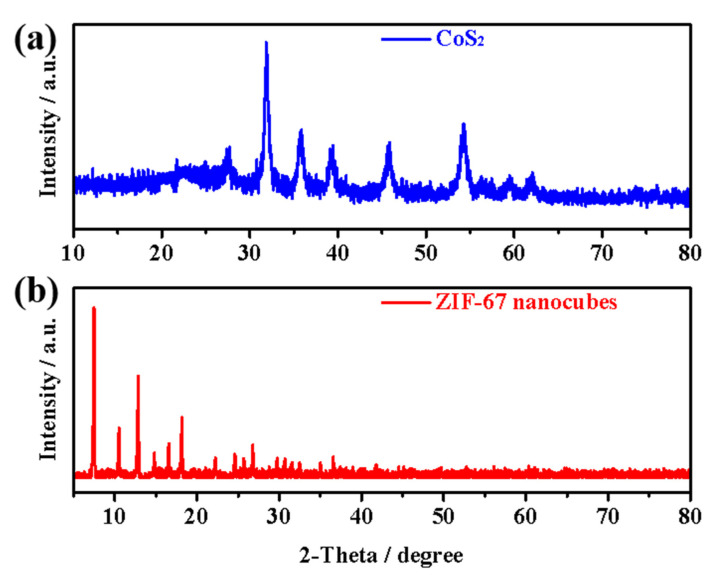
XRD patterns of the samples (**a**) CoS_2_ hollow nanoboxes and (**b**) solid ZIF-67 precursor.

The crystalline structure and morphology of as-obtained solid ZIF-67 precursor and CoS_2_ hollow nanoboxes were examined by powder X-ray diffraction (XRD) spectra, field emission scanning electron microscopy (FESEM) and transmission electron microscopy (TEM). As shown in Figure 2b, the crystalline structure of these as-prepared ZIF-67 assemblies is primarily investigated and the characteristic diffraction peaks in the 2θ rage of 5–40° are found to be precisely matched with the standard solid ZIF-67 precursor without any additional peaks [26,27]. After ion-exchange process, XRD curve showed new diffraction peaks located at 27.9°, 32.3°, 36.3°, 39.8°, 46.3°, 54.9°, 57.6°, 60.2°, and 62.7°, which could be assigned to the (111), (200), (210), (211), (220), (311), (222), (230), (321) planes of CoS_2_ (PDF JCPDS No. 41-1471) [28,29]. Remarkably, XRD patterns indicated the highly crystalline and high purity of the samples as well as verified the successful transformation from solid nanocubes to CoS_2_ hollow nanoboxes during in situ sulfidation.

**Figure 3:**
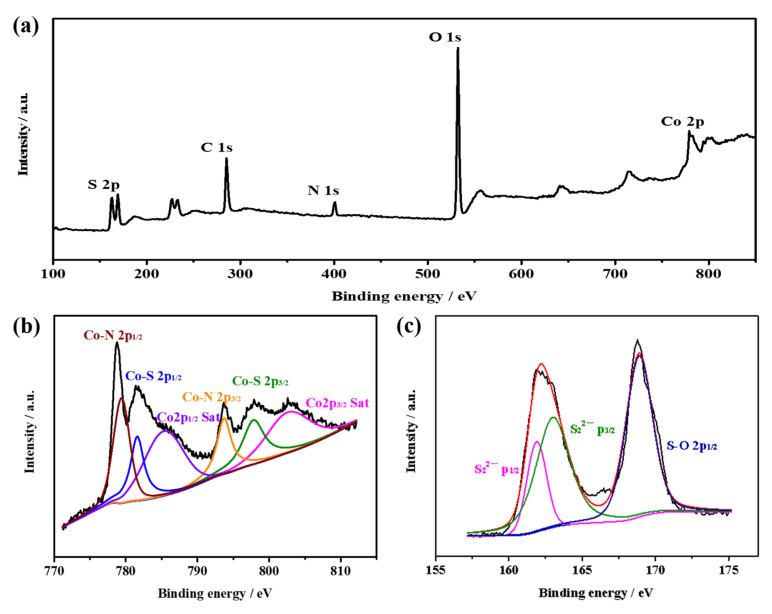
The wide scan XPS spectra of CoS_2_ hollow nanoboxes (**a**) and the high-resolution Co 2p (**b**) and S 2p (**c**) spectrum.

The surface chemical composition of the samples were identified by X-ray photoelectron spectroscopy (XPS) (Figure 3). In the high-resolution Co 2p spectrum (Figure 3b), the two peaks centered at 780.5 eV and 796.9 eV corresponded to the Co 2p_3/2_ and Co 2p_1/2_ of Co^2+^ state, respectively, implying the existence of Co-S bond in the CoS_2_ nanoboxes. Furthermore, the other two peaks located at 802.1 eV and 784.3 eV suggested the existence of N-Co bond due to the insufficient sulfurization of ZIF-67 precursor. In Figure 3c, the characteristic peaks of the S 2p spectrum at 162.2 eV and 163.3 eV were ascribed to the disulfide S_2_^2−^, respectively.

**Figure 4:**
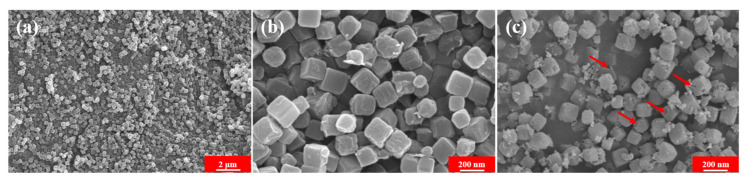
SEM images of solid ZIF-67 precursor (**a**,**b**) and CoS_2_ hollow nanoboxes (**c**).

**Figure 5:**
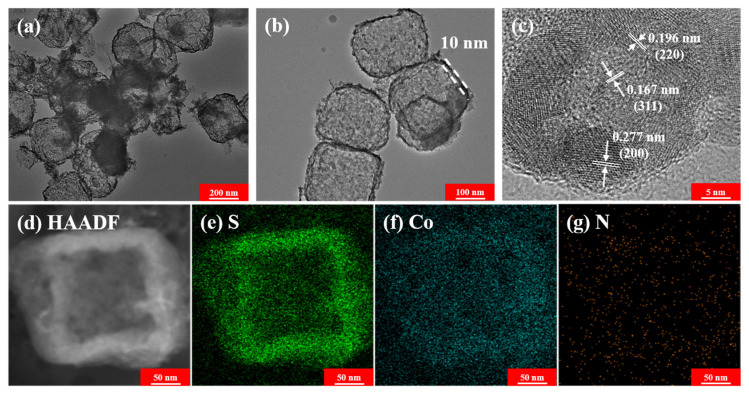
TEM images (**a**,**b**), HRTEM image (**c**), HAADF image and the element distribution maps of the CoS_2_ hollow nanoboxes (**d**–**g**).

The representative SEM images elucidated the morphology and microstructures of ZIF-67 precursor and as-obtained CoS_2_ hollow nanoboxes. As shown in Figure 4a,b, the as-prepared ZIF-67 precursor show well-defined monodisperse solid cube morphology structure with smooth surface and relatively narrow size distribution, and the average particle size was around 200 nm (Figure 4a,b). After in situ ion-exchange process in the presence of CS_2_, the amounts of CS_2_ and the reaction temperatures have great influence on the morphology: high dosage and high reaction temperature will destroy its structure (see Supplementary Figure S1). When the dosage of CS_2_ is 0.3 mL and the reaction temperature was 140 °C, the cube structure could be maintained (Figure 4c) without suffering severe damage and the partially broken shell shown in Figure 4c (see the red arrows) revealed that the as-prepared nanoboxes had a hollow structure. Meanwhile, compared with pristine ZIF-67 precursor, the surface of CoS_2_ hollow nanoboxes became wrinkled and rough due to the successful sulfidation. TEM and high resolution (HRTEM) images further corroborated the well-defined hollow structure (Figure 5a,b). The TEM images (Figure 5a,b) clearly showed that the composites displayed unique hollow structure and held uniform shell with the thickness of 10 nm and a cavity size of 180 nm. As shown in Figure 5c, the interplaner spacing (see the white lines in the Figure 5c) of 0.167 nm, 0.196 nm and 0.277 nm could be assigned to the (311), (220) and (200) plane of the CoS_2_ hollow nanoboxes, respectively. HAADF and the element mappings images in Figure 5d–g further verify the homogeneous distribution of Co and S elements in the hollow nanoboxes.

**Figure 6:**
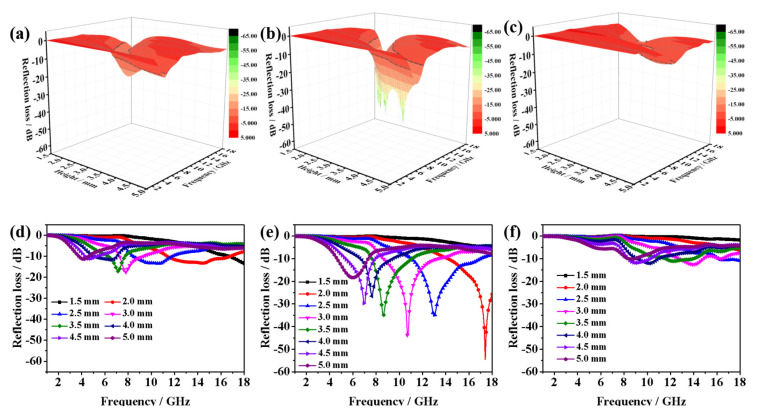
Three dimensional reflection loss values of the CoS_2_/paraffin composites with different contents under varied thicknesses (1.5–5.0 mm) in the frequency range of 1–18 GHz: (**a**,**d**) 10 wt%, (**b**,**e**) 20 wt%, (**c**,**f**) 30 wt%.

**Figure 7:**
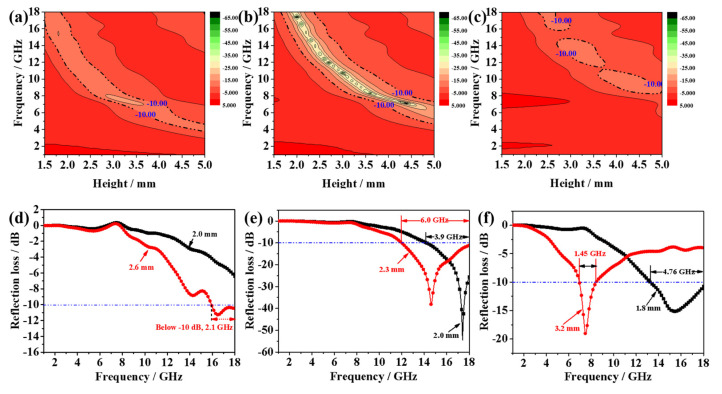
Color map of the reflection loss values and effective absorption bandwidth CoS_2_/paraffin composites with different CoS_2_ proportions at different thicknesses: (**a**,**d**) 10 wt%, (**b**,**e**) 20 wt%, (**c**,**f**) 30 wt%. (The area enclosed by the dotted lines represents the area of effective absorption below—10 dB).

Generally, the minimum reflection loss (*RL*min), effective absorption bandwidth (EAB) and absorbent matching thickness (dm) are the main factors influencing the microwave absorption performance of absorbing materials. The effective absorption bandwidth refers to the reflection loss value below −10 dB, which is equal to 90% of the incident electromagnetic wave be attenuated. According to transmission line theory, the reflection loss values of absorbing materials can be calculated by the following formula [1,2,30,31,32,33]:

(1) $$RL(dB)=20\log\left|\left(Z_{\mathrm{in}}-Z_{0}\right)/\left(Z_{\mathrm{in}}+Z_{0}\right)\right|$$

(2) $$Z_{\mathrm{in}}=Z_{0}{\left(\frac{\mu_{r}}{\varepsilon_{r}}\right)}^{1/2}\tanh\left[j\left(2\pi fd/c\right){\left(\varepsilon_{r}\mu_{r}\right)}^{1/2}\right]$$

where *Z*_in_, *Z*_0_, *μ_r_*, *ε_r_*, *f*, *d* and *c* are the input impedance of the absorber, the impedance of free space, relative complex permeability and relative complex, the microwave frequency, the thickness of the absorber and the velocity of light, respectively. Taking the practical applications into consideration, the filling contents ratio is an important index to evaluate the microwave absorption performance of wax-based absorbents. To investigate the microwave absorption performance of CoS_2_ hollow nanoboxes, various filling contents of the products were mixed with paraffin to form composites by a simple blending method. Figure 6 and Figure 7 exhibit the 3D presentations of calculated theoretical *RLs* and *RL* curves of the as-obtained hollow CoS_2_/paraffin composites with different thicknesses (1.5–5 mm) in 1–18 GHz at various filler loadings. When the filling content of CoS_2_ hollow nanoboxes was 10 wt%, the minimum *RL* value was −12.65 dB at 13.84 GHz with an optimum matching thickness of 3.1 mm (Figure 6a,d). It is notable that the filling contents of CoS_2_ hollow nanoboxes reached to 20 wt%, and that the microwave absorption performance of hollow CoS_2_/paraffin composites has been enhanced obviously. The *RL*min value reached as strong as −54.48 dB at 17.4 GHz and the maximum absorption bandwidth reached 6.0 GHz with a thin thickness of 2.3 mm (Figure 6b,e and Figure 7b,e), which indicates that the *RL*min value and the EAB are much larger and broader compared with the other samples. After further increase in the filling contents to 30 wt%, the absorption performance deteriorated (Figure 6c,f and Figure 7c,f), much less than that of 20 wt%, implying that the filling contents and the thickness of the absorbers have a vital influence on the microwave absorption ability.

**Figure 8:**
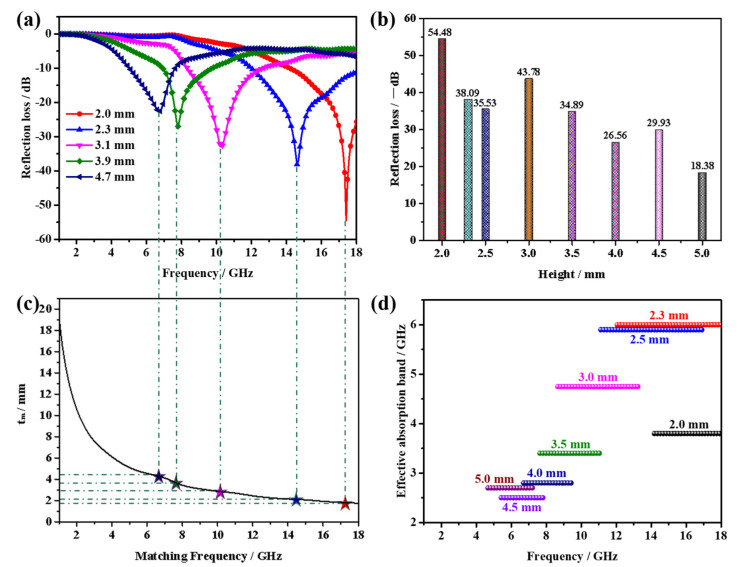
(**a**) Reflection loss curves of different thicknesses, (**b**) minimum reflection loss values of different thicknesses, (**c**) dependence of matching thickness (t_m_) on matching frequency (f_m_) at the wavelength of 1/4, and (**d**) Effective absorption band at different thicknesses of paraffin-based composites containing 20 wt.% CoS_2_ hollow nanoboxes.

To embody the excellent microwave absorption performance of CoS_2_ hollow nanoboxes in detail, related graphs are shown in Figure 8. The reflection loss of the 20 wt% samples differ with the layer thickness (Figure 8a). We conducted a comparison of the RL values and EAB for various layer thicknesses, as depicted in Figure 8b,d. It is concluded that 20 wt% samples still have a rather attractive performance in MA even if at a single matching thickness. Nevertheless, what is noteworthy is the RL peaks gradually shift towards a low frequency with increasing thickness (Figure 8a). This phenomenon can be explained by the quarter-wavelength cancellation theory. To be more specific, the minimum RL value is achieved when the matching thickness and matching frequency satisfy the following equation [34,35,36]:

(3) $$t_{m}=\frac{n\lambda }{4}=\frac{nc}{4f_{m}\sqrt{\left|\varepsilon_{r}\right|\left|\mu_{r}\right|}}\begin{array}{ccc} & & \left(n=1,3,5\ldots \ldots \right) \end{array}$$

As we have seen in Figure 8c, the extension cords of different matching thickness and corresponding peaks intersect at the point located around the *λ*/4 curve. Consequently, the matching thickness and peak frequency for microwave absorption by 20 wt% samples obey the quarter-wavelength cancellation model.

**Figure 9:**
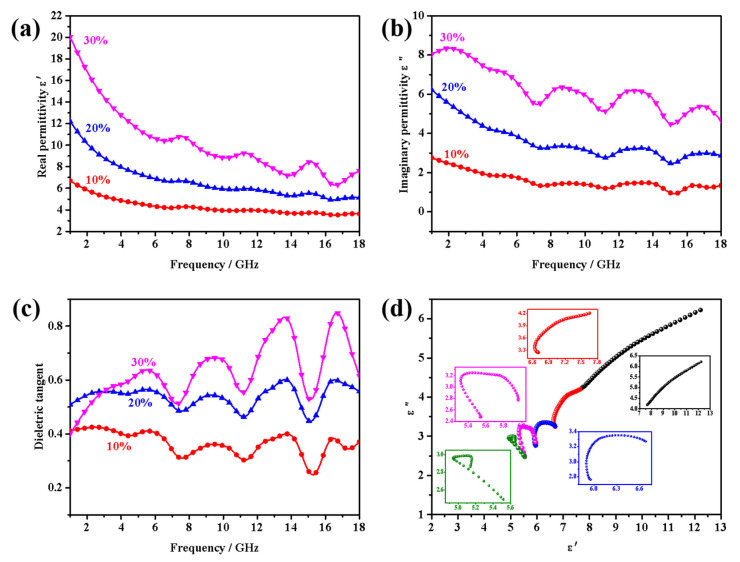
Frequency dependence of the real and imaginary parts of complex permittivity (**a**,**b**) dielectric loss tangent (**c**) of different samples and the Cole–Cole plots of samples with mass fraction of 20 wt% (**d**).

It is well known that the microwave absorption properties of absorbing materials mainly depend on the complex permittivity (*ε*_r_ = *ε*′ − j*ε*″) and complex permeability (*μ*_r_ = *μ*′ − j*μ*″), where the real parts (*ε*′ and *μ*′) stand for the storage capacity of electrical and magnetic energy and the imaginary parts (*ε*″ and *μ*″) represent the decay of the electric and magnetic energy of the material, respectively [37,38,39,40]. Therefore, the higher *ε*″ and *μ*″ values could endow the better microwave absorption properties. In order to better explore the associated microwave absorbing mechanism of CoS_2_ hollow nanoboxes, the frequency dependence relative permittivity for CoS_2_/paraffin with various filling contents is depicted in Figure 9. Over the whole frequency range, the values of *ε*′ and *ε*″ increase with the increase in the filling contents and the composites containing 30 wt% CoS_2_ hollow nanoboxes have much higher *ε*′ and *ε*″ values than those of the other two samples, which could endow them with better microwave absorption performance. Nevertheless, their absorbing performance is weaker than that of samples with 20 wt% filling contents, which may be due to their poor impedance matching performance. The *ε*′ and *ε*″ values display a downward trend in the frequency range of 2–18 GHz, though a vigorous fluctuation occurred at 7–18 GHz in *ε*″ curve of all composite samples. Such a phenomenon existed in many pristine absorbing materials and most composites can be explained by frequency dispersion behavior. The multiple resonance peaks founded in the *ε*″ curves of all samples maybe correlated with the interfaces, which would cause the displacement current lag. According to the Debye theory and free electron theory, it is deduced that dipolar polarization triggered by nitrogen atoms and defects in materials, interfacial polarization originating in various interfaces, and metal sulfide materials’ inherent conductivity loss together give rise to dielectric loss. Commonly, the relaxation process has a significant influence on the dielectric loss of microwave absorption materials, which can be explained by a Cole–Cole semicircle. According to the Debye dipolar relaxation, the relative complex permittivity (*ε*_r_) can be described by the following equation [37,38]:

(4) $$\varepsilon_{r}=\varepsilon^{\prime }-j\varepsilon^{\prime \prime }=\varepsilon_{\infty }+\frac{\varepsilon_{s}-\varepsilon_{\infty }}{1+j2\pi f\tau }$$

where *ε*_s_ is the static permittivity; *τ* and *ε*_∞_ are the polarization relaxation time, and relative dielectric permittivity at the high-frequency limit, respectively. From Equation (4), it can be deduced as shown below

(5) $$\varepsilon^{\prime }=\varepsilon_{\infty }+\frac{\varepsilon_{s}-\varepsilon_{\infty }}{1+{\left(2\pi f\right)}^{2}\tau^{2}}$$

(6) $$\varepsilon^{\prime \prime }=\frac{2\pi f\tau (\varepsilon_{s}-\varepsilon_{\infty })}{1+{\left(2\pi f\right)}^{2}\tau^{2}}$$

According to Equations (5) and (6), the final equation indicating the relationship between *ε*′ and *ε*″ can be deduced

(7) $${\left(\varepsilon^{\prime }-\frac{\varepsilon_{s}+\varepsilon_{\infty }}{2}\right)}^{2}+{\left(\varepsilon^{\prime \prime }\right)}^{2}={\left(\frac{\varepsilon_{s}-\varepsilon_{\infty }}{2}\right)}^{2}$$

Thus, the curve of ε′ versus ε″ is a single semicircle, which is named a Cole–Cole semicircle, and one relaxation process usually corresponds to one semicircle. Curves of *ε*′ versus *ε*″ for 20 wt% samples are portrayed in Figure 9d, where five Cole–Cole semicircles can be observed. Such a curve indicates there are several continuous strong Debye polarization relaxation processes, which is entirely in accord with the resonance phenomena of *ε*′′ curve. However, without ignorance, the Cole–Cole semicircles are distorted, indicating there might be other mechanisms.

In addition to dielectric loss, the presence of CoS_2_ hollow nanoboxes could produce weak magnetic loss, which contributed to the improvement of the microwave absorption performance to some degree. Available literature sources show that the magnetic loss comes from hysteresis loss, domain-wall resonance, eddy current resonance, natural resonance and exchange resonance [39]. In consideration of the restricted conditions of magnetic hysteresis and domain wall resonance, which are only generated in a strong magnetic field and only existed in the low frequency (<2 GHz), respectively, eddy current loss, natural resonance and exchange resonance have important roles in the microwave absorption. Eddy current effect hinders the electromagnetic wave entering the absorber, which should be suppressed. The eddy current can be calculated with the following eddy equation [40]:

(8) $$C_{0}=\mu^{\prime \prime }{\left(\mu^{\prime }\right)}^{-2}f^{-1}=2\pi \mu_{0}\sigma d^{2}/3$$

where *μ*_0_ is the permeability in vacuum, *σ* is the conductivity of microspheres and *d* is the thickness. Based on the Equation (7), if *C*_0_ is a constant, the existence of eddy current effect would be identified. From the curves of *μ*″(*μ*′)^−2^ f ^−1^ against frequency shown in Figure S2 (see Supporting Information, Supplementary Figure S2), it is found that all the curves of composites have obvious fluctuations in the frequency range from 1 to 18 GHz, resulting in excluding the possible eddy current effect. Therefore, the magnetic loss is mainly derived from natural resonance in low frequency and exchange resonance in high frequency. The natural resonance can be assessed by the following equations [32]:

(9) $$f_{r}=rH_{\alpha }/2\pi$$

(10) $$H_{\alpha }=4\left|K_{1}\right|/3\mu_{0}M_{s}$$

where the *f*_r_, *r*, *H*_α_, |*K*_1_| and *M*_s_ are defined as the frequency of natural resonance, gyromagnetic ratio, anisotropic energy, anisotropic coefficient and saturation magnetization, respectively. The natural resonance can be influenced by two aspects according to the natural-resonance equation. On the one hand, the smaller size of magnetic cores gives rise to the increase in anisotropic energy of the composite due to the surface anisotropic field by the reduced size effect. On the other hand, the lower *M*_s_ value of the weak magnetic CoS_2_ will increase with higher anisotropic energy. As is recognized, the higher anisotropic energy is beneficial to enhance microwave absorption properties. Furthermore, the peaks at high frequency can be ascribed to the exchange resonance.

Electromagnetic wave reflection usually includes surface reflection and multiple reflection, in which multiple reflections are usually caused by scattering effects due to inhomogeneity within the material internal. As we all know, adjusting and designing porous and hollow structures can realize multiple reflections, thus effectively extending the electromagnetic wave propagation path. Furthermore, the presence of hollow structures can regulate electromagnetic parameters to achieve impedance matching characteristics, improving the electromagnetic wave absorption of materials’ performance. The impedance matching ratio (*Z*) and attenuation constant (*α*) of absorbing materials can be calculated by the following formulas, respectively [41,42].

(11) $$Z=\left|Z_{\mathrm{in}}/Z_{0}\right|=\sqrt{\left|\mu_{r}/\varepsilon_{r}\right|}\tanh\left[j\left(2\pi fdc\right)\sqrt{\mu_{r}\varepsilon_{r}}\right]$$

(12) $$\alpha =\frac{\sqrt{2}\pi f}{c}\sqrt{\left(\mu^{\prime \prime }\varepsilon^{\prime \prime }-\mu^{\prime }\varepsilon^{\prime }\right)+\sqrt{{\left(\mu^{\prime \prime }\varepsilon^{\prime \prime }-\mu^{\prime }\varepsilon^{\prime }\right)}^{2}+{\left(\mu^{\prime }\varepsilon^{\prime \prime }-\mu^{\prime \prime }\varepsilon^{\prime }\right)}^{2}}}$$

**Figure 10:**
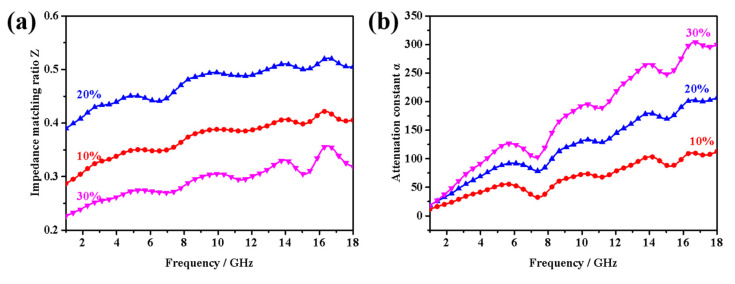
(**a**) The impedance matching ratio Z and (**b**) attenuation constant α of the CoS_2_/paraffin composites with different filling contents.

As presented in Figure 10, it is noted that the samples with 30 wt% filler loading have the largest α values among three samples, indicating an ideal attenuation effect for the incident microwave. However, due to their poor impedance matching performance, most of the incident microwave will reflect at the air-absorber interface rather than enter into the absorber. In comparison, the samples with 20 wt% filler loading show both the best impedance matching performance and excellent attenuation performance, resulting in the best microwave absorption performance, which is consistent with the previous discussion of electromagnetic parameters.

## 4. Conclusions

In summary, the CoS_2_ hollow nanoboxes have been successfully fabricated by the in situ ion-exchange process. The amounts of CS_2_ and the reaction temperature as well as reaction time play an important role in hollow nanoboxes’ structure during the ion-exchange process. The microwave absorption performance of different contents of paraffin-based composites was investigated in detailed. The results demonstrate that paraffin-based CoS_2_ composites with 20 wt.% filling contents show significantly enhanced microwave absorption performance with *RL_min_* of up to −54.48 dB at 17.4 GHz and the best effective absorption bandwidth of 6.0 GHz (*RL* ≤ −10 dB) due to their special hollow nanoboxes structure and good impedance matching. The unique hollow structure contributes to the multiple reflection of electromagnetic wave and could regulate the electromagnetic parameters.

## Data Availability

Not applicable.

## Supplementary Materials

The following supporting information can be downloaded at: https://www.mdpi.com/2079-4991/12/16/2876/s1, Figure S1: The as-obtained products of different CS2 dosage and reaction temperatures. (**a**) 160 °C–0.3 mL CS2 (**b**) 160 °C–0.5 mL CS2 (**c**) 180 °C–0.3 mL CS2. Figure S2: Frequency dependence of the real and imaginary parts of complex permeability and C_0_.
