# Supplementary material for: In Situ Formation of CoS2 Hollow Nanoboxes via Ion-Exchange for High-Performance Microwave Absorption
Source: Nanomaterials (Basel). 2022 Aug 21;12(16):2876. doi: 10.3390/nano12162876 (PMC9460408; doi:10.3390/nano12162876)
Supplement: Supplementary file 1 [file nanomaterials-12-02876-s001.zip › nanomaterials-1851837-supplementary.pdf]

In Situ Formation of CoS_2 Hollow Nanoboxes via Ion-exchang for High-Performance Microwave Absorption

Dongwei Xu [a*], Huanhuan Guo [a], Feifan Zhang [a], Yanmei Wu [a], Xiaoqin Guo [a,], Yumei Ren [a*], Desheng Feng [a]

[a] School of Material Science and Engineering, Henan Key Laboratory of Aeronautical Materials and Application Technology, Zhengzhou University of Aeronautics, Zhengzhou, Henan 450046, China

* Corresponding author:

Dr.Dongwei Xu, E-mail:xudongwei1029@126.com

Dr.Yumei Ren, E-mail:ymren@zua.edu.cn

Supplementary Figures:

Figure S1. The as-obtained products of different CS_2 dosage and reaction temperatures：(a) 160 ℃-0.3 mL CS_2 (b) 160 ℃-0.5 mL CS_2 (c) 180 ℃-0.3 mL CS_2.

Figure S2. Frequency dependence of the real and imaginary parts of complex permeability and C_0

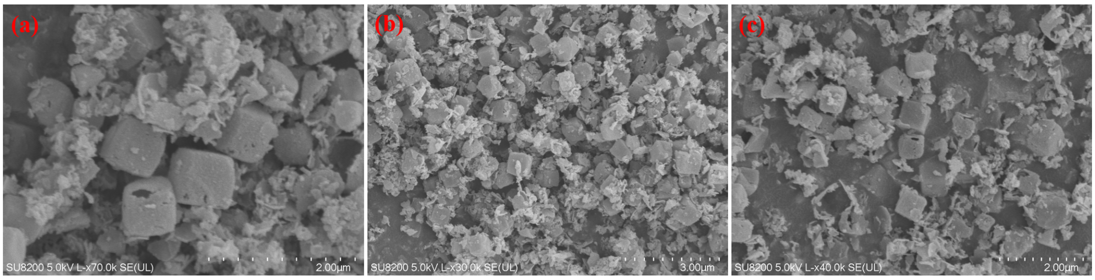

Figure S1

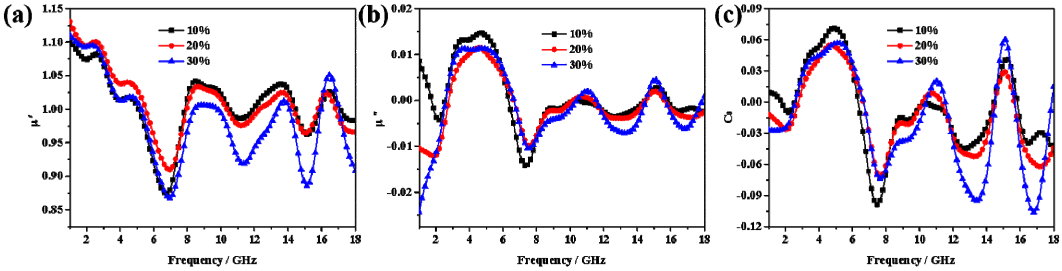

Figure S2
